# Protocol for the recovery and deep sequencing of short ssDNA pools from transient, fuel-dependent coacervate droplets

**DOI:** 10.1016/j.xpro.2025.104293

**Published:** 2025-12-23

**Authors:** Anna-Lena Holtmannspötter, Corbin Machatzke, Job Boekhoven, Hannes Mutschler

**Affiliations:** 1 TU Dortmund University, Otto-Hahn-Strasse 4a, 44227 Dortmund, Germany; 2 Department of Bioscience, School of Natural Sciences, Technical University of Munich, Lichtenbergstrasse 4, 85748 Garching, Germany

**Keywords:** sequence analysis, sequencing, chemistry

## Abstract

Complex coacervate droplets are synthetic cell models that sequester nucleic acids. Here, we present a protocol for the recovery and deep sequencing of single-stranded DNA (ssDNA) from metabolically active coacervate droplets. We describe time-resolved harvesting, ssDNA recovery, and sequence-independent library preparation for next generation sequencing (NGS), along with an analysis pipeline to assess enrichment dynamics and sequence distributions. The protocol is adaptable to diverse droplet systems.

For complete details on the use and execution of this protocol, please refer to Machatzke et al.^1^

## Highlights

• Protocol to prepare and harvest ssDNA from fueled complex coacervates
• Sequence-independent, orientation-specific ssDNA library preparation for NGS
• Comprehensive data analysis pipeline to standardize results across research groups

## Abstract (editor highlights)

Publisher’s note: Undertaking any experimental protocol requires adherence to local institutional guidelines for laboratory safety and ethics.

## Graphical abstract

**Figure:**
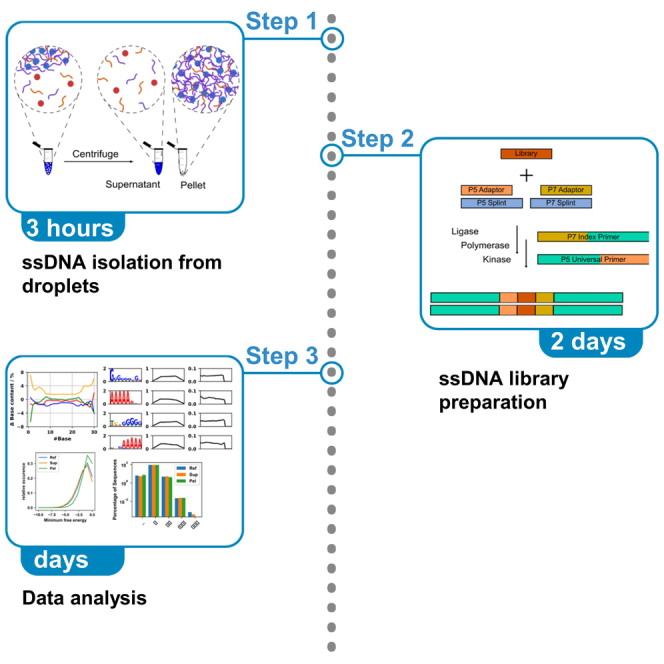

## Teaser

Complex coacervate droplets are synthetic cell models that sequester nucleic acids. Here, we present a protocol for the recovery and deep sequencing of single-stranded DNA (ssDNA) from metabolically active coacervate droplets. We describe time-resolved harvesting, ssDNA recovery, and sequence-independent library preparation for NGS, along with an analysis pipeline to assess enrichment dynamics and sequence distributions. The protocol is adaptable to diverse droplet systems.

## Before you begin

### Rational for method development

We recently developed fuel-dependent complex coacervate droplets sustained by a chemical reaction cycle.^2^^,^^3^ These non-equilibrium compartments require continuous fuel to form and persist, and will decay without it.^4^ Under defined conditions, they can undergo growth-division cycles.^5^ While they provide a powerful platform to study life-like, out-of-equilibrium behavior, these droplets lack a heritable genotype, which would be required for them to undergo Darwinian evolution. We envision that self-replicating DNA sequences could serve as a genotype if sequence-dependent interactions modulate droplet properties.^6^ To test this hypothesis, we established a deep-sequencing workflow that links DNA sequence to droplet-level phenotypes, enabling high-throughput mapping of sequences that bias droplet formation, stability and dynamics.

Specifically, we developed a protocol to retrieve, identify, and analyze ssDNA molecules that partition into the polymer-rich phase of a transient, fuel-driven complex coacervate system and which ssDNA molecules remain in the polymer-poor phase. Because sequence determinants of partitioning are difficult to predict, this protocol enables experimental determination of enrichment and depletion rules and dynamics.^7^^,^^8^^,^^9^ The workflow is compatible with other synthetic and protocellular model systems, provided that ssDNA recovery and library preparation are adjusted accordingly. The protocol can also be adapted to RNA-based systems, if the extraction is modified and strand-aware library steps follow cDNA by reverse transcription. After ssDNA recovery, we present a strand-specific, sequence-agnostic library preparation method for deep sequencing. Finally, we provide a modular analysis pipeline tailored to short (e.g., prebiotically plausible) sequences,^10^^,^^11^^,^^12^^,^^13^ quantifying nucleotide composition, predicted intramolecular structure, and motif enrichment.

### Innovation

We have developed a method for the time-resolved recovery and deep sequencing of ssDNA from transient, fuel-driven complex coacervate droplets.^2^^,^^3^ Because these droplets have limited lifetimes, we have implemented a rapid quenching and harvesting protocol to capture polymer-rich and polymer-poor sequence pools with minimal bias. To generate the deep-sequencing library, we adapted the SRSLY (single reaction single-stranded library) protocol^14^ for use with low amounts of unknown ssDNA input, thereby increasing yields while preserving strand orientation and minimizing sequence bias.

On the computational side, we offer a modular and customizable analysis pipeline tailored to short oligonucleotides, typical of synthetic coacervate droplets (∼10–100 nt). The modules report quality metrics, nucleotide composition, intramolecular folding, and motif discovery. The pipeline also promotes standardization across laboratories working on synthetic/protocell systems and can be extended to alternative nucleic acid backbones such as RNA with minimal changes to recovery and library preparation.

Taken together, the protocol provides a reproducible end-to-end workflow for identifying and analyzing sequence determinants of partitioning into static and metabolically active droplet systems, as well as related compartments.

### Buffer preparation

**Timing: 30 min to 1 h**

1. Prepare 500 mM MES pH 5.3 stock solution.
  a. Dissolve 53.3 g MES monohydrate in 400 mL nuclease-free ddH_2_O.
  b. Adjust the pH to 5.3, then top up to 500 mL and mix thoroughly before use.
2. Prepare a 41 mM polyU (charge concentration/monomer concentration) stock solution.
  a. Dissolve 14.15 mg polyuridylic acid potassium salt in 1 mL nuclease-free ddH_2_O.
3. Prepare 155 mM RG_3_ peptide stock solution.
  a. Dissolve 500 mg Ac-FRGRGRGD in 1 mL nuclease-free ddH_2_O.
  b. Adjust pH with 1 M NaOH to pH 5.3 (∼475 μL).
  c. Add nuclease-free ddH_2_O until the total volume is equal to 2.475 mL.

**CRITICAL:** Do not prepare a stock solution with a peptide concentration that is too high! If the concentration is too high, the peptide will precipitate over time.

**CRITICAL:** Immediately adjust the pH of the peptide solution.

**CRITICAL:** Always verify the quality of custom peptides. Identity and purity can be confirmed by mass spectrometry and HPLC. Demonstrate correct formation of complex coacervates with no significant precipitation under the conditions described in the detailed protocol.

**CRITICAL:** Always store RNA oligonucleotides at −20°C or −80°C and thaw them on ice. If not stored properly, RNA can hydrolyze, leading to insufficient droplet formation.

4. Prepare a 5 M NaCl stock solution.
  a. Dissolve 29.22 g NaCl in 70 mL nuclease-free ddH_2_O.
  b. Adjust volume to 100 mL with nuclease-free ddH_2_O after complete dissolution.
5. Prepare 400 mM EDC stock solution.
  a. Dissolve 19.2 mg EDC in 230 μL nuclease-free ddH_2_O.

**CRITICAL:** Always prepare the EDC solution fresh. Ideally, EDC should be stored at −20°C under an inert gas.

6. Prepare 50% w/v PEG-8000 stock solution.
  a. Dissolve 20 g of PEG-8000 in 23 mL of nuclease-free ddH_2_O.
  b. Adjust volume to 40 mL with nuclease-free ddH_2_O after complete dissolution.

***Note:*** To dissolve PEG-8000, apply slight heat while stirring constantly. If it does not fully dissolve, add small amounts of nuclease-free ddH_2_O, ensuring that the total volume does not exceed 40 mL.

**Table:** Table specifying buffer storage conditions and minimum shelf-life

Buffer	Storage conditions	Minimum shelf-life
MES buffer	18°C–27°C	Indefinitely
polyU stock	−20°C	6–12 months
Peptide stock	4°C	6–12 months
NaCl stock	18°C–27°C	Indefinitely
EDC stock	On ice	2–6 hours
PEG-8000 stock	18°C–27°C	Indefinitely

## Key resources table

**Table:** 

REAGENT or RESOURCE	SOURCE	IDENTIFIER
**Chemicals, peptides, and recombinant proteins**		

RG_3_ peptide (Ac-FRGRGRGD), >98%	Caslo	N/A
MES monohydrate	AppliChem	Cat# A1074,0500
Polyuridylic acid potassium salt	Sigma-Aldrich	Cat# P9528-25MG
Sodium Chloride	Sigma-Aldrich	Cat# S9888-2.5KG-M
1-ethyl-3-(3-dimethylaminopropyl) carbodiimide (EDC)	Sigma-Aldrich	Cat# E6383-5G
RNase Cocktail	Invitrogen	Cat# AM2286
PEG-8000	Carl Roth	Cat# 0263.1
T4 DNA ligase	New England Biolabs	Cat# M0202S
T4 Polynucleotide Kinase (PNK)	New England Biolabs	Cat# M0201S
NEBNext Ultra II Q5 Master Mix	New England Biolabs	Cat# M0544L

**Critical commercial assays**		

NEBNext Multiplex Oligos for Illumina (Index Primer Set)	New England Biolabs	Eg. E7500L or E7710L
Monarch Spin PCR & DNA Cleanup Kit	New England Biolabs	T1130L
NucleoMag NGS Clean-up and Size Selection	Macherey-Nagel	Ref. 744970.5
PhiX Control v3	Illumina	15017666

**Oligonucleotides**		

N30 library: NNNNNNNNNNNNNNNNNNNNNNNNNNNNNN	Integrated DNA Technologies	N/A
SRSLY P5 adapter:/5AmMC12/AC ACT CTT TCC CTA CAC GAC GCT CTT CCG ATC T	Integrated DNA Technologies	N/A
SRSLY P5 splint:/5AmMC6/NN NNN NNA GAT CGG AAG AGC GTC GTG TAG GGA AAG AGT GT/3AmMO/	Integrated DNA Technologies	N/A
SRSLY P7 adapter:/5Phos/AG ATC GGA AGA GCA CAC GTC TGA ACT CCA GTC A/3ddC/	Integrated DNA Technologies	N/A
SRSLY P7 splint:/5AmMC12/GT GAC TGG AGT TCA GAC GTG TGC TCT TCC GAT CTN NNN NNN/3AmMO/	Integrated DNA Technologies	N/A
SRSLY P5 adapter Cy5 tagged (for troubleshooting):/5Cy5/ACA CTC TTT CCC TAC ACG ACG CTC TTC CGA TCT	Integrated DNA Technologies	N/A
SRSLY P7 adapter FAM tagged (for troubleshooting):/5Phos/AG ATC GGA AGA GCA CAC GTC TGA ACT CCA GTC A/36-FAM/	Integrated DNA Technologies	N/A
Tagged RNA:/5Cy5/rArArArArCrCrArGrUrC	Integrated DNA Technologies	N/A

**Software and algorithms**		

Illumina Sequencing Analysis Viewer 2.4.7	Illumina	https://support.illumina.com/sequencing/sequencing_software/sequencing_analysis_viewer_sav.html
FastQC 0.12.1		https://www.bioinformatics.babraham.ac.uk/projects/fastqc/
MultiQC 1.15		https://github.com/MultiQC/MultiQC
Cutadapt 4.3		https://cutadapt.readthedocs.io/en/stable/
Seqkit 2.4.0		https://bioinf.shenwei.me/seqkit/
Vienna RNA package, RNAfold 2.4.17		https://www.tbi.univie.ac.at/RNA/
RNAshapes 3.4		https://bibiserv.cebitec.uni-bielefeld.de/rnashapes
Python 3.8.8		https://www.python.org/
Meme Suite, streme 5.5.7		https://meme-suite.org/meme/

**Other**		

4150 TapeStation	Agilent	https://www.agilent.com/en/product/automated-electrophoresis/tapestation-systems/tapestation-instruments/4150-tapestation-system-297322
Illumina iSeq 100	Illumina	https://www.illumina.com/systems/sequencing-platforms/iseq.html
ProFlex PCR Systems or equivalent thermocycler	Thermo Fisher Scientific	Cat# 4483636
Micro Star 17R or equivalent centrifuge	VWR	Cat# 521-1647
DynaMag-2 or equivalent magnetic rack	Thermo Fisher Scientific	Cat# 12321D
SafeSeal reaction tubes or similar low binding reaction tubes	Sarstedt	Cat# 72.704.700

## Step-by-step method details

### Droplet preparation and DNA recovery

**Timing: 3 h**

First, we describe how to prepare fuel-dependent complex coacervates in the presence of an ssDNA library. Then, we explain how to separate the phases and recover the ssDNA for downstream analysis.

1. Prepare complex coacervates and separate phases.
  a. Thaw ssDNA library (and tagged RNA, if used) on ice.
  b. Prepare complex coacervates mix (900 μL).

**Table:** 

Reagent	Stock	Final	V/μL
MES pH 5.3	500 mM	200 mM	400
RG3 peptide	155 mM	23 mM	150
polyU	41 mM	4.1 mM	100
N30 library	3 μg/μL	9 μg	3
Tagged RNA (optional)	100 μM	1 μM	10
ddH_2_O			237 or 247
    ***Note:*** Adding tagged RNA makes the pellet easier to detect after centrifugation. While this is not strictly necessary, it is recommended for initial trials. The RNA sequence is not critical, as the RNase cocktail will remove all RNAs.
  c. Add 100 μL 400 mM EDC solution to the complex coacervates master mix. Mix thoroughly by pipetting up and down.
    ***Note:*** Within seconds of mixing the sample with fuel, the solution should become visibly turbid, indicating coacervate formation. If this does not occur and the sample remains clear, refer to problem 1. For representative static images and time-lapse videos of the expected turbidity and droplet morphology, see Donau et al., 2020.^2^
    **CRITICAL:** From the addition of EDC until the supernatant is removed, it is crucial to adhere to all incubation times precisely and work quickly. The fuel-dependent system has a very short lifespan.
  d. Incubate for 1 minute at ∼25°C.
  e. Spin for 3 minutes at 16.000 g in a centrifuge set to 25°C.
    **CRITICAL:** The temperature should not exceed 30°C during centrifugation. The lifetime of droplets is greatly affected by temperature. Lower temperatures may greatly extend lifetimes, whereas higher temperatures can drastically decrease them.
  f. After centrifugation, immediately remove all supernatant very thoroughly and store it for downstream analysis.
    ***Note:*** Use a P1000 to remove most of the bulk of the supernatant without disturbing the pellet. Then, using a P10, remove as much liquid as possible from the pellet. Discard these small volumes.
  g. Resuspend the pellet in 10 μL 5 M NaCl by pipetting up and down.
  h. After resuspension, the solution should be clear. Add 40 μL ddH_2_O.
  i. Use the Monarch Spin PCR & DNA Cleanup Kit according to the manufacturer’s instructions for the “small oligo” protocol to clean up 50 μL of pellet and 50 μL of supernatant:
    i. Add 100 μL Buffer BZ to the solution.
    ii. Add 300 μL Isopropanol. Mix well by pipetting up and down. Do not vortex.
    iii. Transfer 450 μL solution onto a spin column and spin for 1 minute at 16,000 g. Discard flow-through.
    iv. Add 500 μL Buffer WZ to the spin column and spin for 1 minute at 16,000 g. Discard flow-through.
    v. Repeat step iv.
    vi. Transfer the spin column to a fresh 1.5 mL sample tube. Spin empty for 1 minute at 16.000 g to remove residual buffer.
    vii. Transfer the spin column to a fresh sample tube. Add 10 μL ddH_2_O to the center of the matrix to elute DNA. Spin for 1 minute at 16,000 g, then discard the spin column.
2. Digest polyU RNA from both samples.
  a. Add 1 μL RNase cocktail to both samples.
    ***Note:*** Always keep enzyme mixes in −20°C cooling blocks.
  b. Incubate the samples in a thermocycler for 2 hours at 37°C, followed by 10 minutes at 65°C with the lid set to 105°C.
    ***Note:*** 2 μL of the recovered, RNase-digested ssDNA can be used for analysis via urea-PAGE gel (20% 19:1 acrylamide: bisacrylamide). If low yields are observed in this step, refer to problem 2.

### Library preparation and sequencing

**Timing: 2 days**

This step describes how to prepare ssDNA for sequencing using sequence-agnostic, orientation-specific splinted ligations. We then provide a brief overview of how to perform sequencing using an iSeq 100. However, any Illumina-type sequencer that is compatible with the adapters can be used. If you are proceeding with in-house sequencing, remember to thaw the sequencing reagents according to the manufacturer’s instructions. This process usually takes 9–18 hours.

3. Ligation of isolated ssDNA to the adapter oligonucleotides via splinted ligation.
  a. Thaw adapter and splint oligos on ice.
  b. Prepare 10 μL of P5 adapter and splint mix per ssDNA sample.

**Table:** 

Reagent	Stock	Final	V/μL
T4 DNA Ligase reaction buffer	10×	1×	1
SRSLY P5 adapter	100 μM	10 μM	1
SRSLY P5 splint	100 μM	20 μM	2
ddH_2_O			6
  c. Prepare 10 μL of P7 adapter and splint mix per ssDNA sample.

**Table:** 

Reagent	Stock	Final	V/μL
T4 DNA Ligase reaction buffer	10×	1×	1
SRSLY P7 adapter	100 μM	10 μM	1
SRSLY P7 splint	100 μM	20 μM	2
ddH_2_O			6
  d. Anneal oligonucleotides by heating the reaction mix to 95°C for 10 seconds and then cooling them down to 10°C at a ramp rate of 0.1°C/s in a thermocycler.
  e. Prepare 78 μL Ligation mix per ssDNA sample. Mix well by pipetting up and down or vortexing.

**Table:** 

Reagent	Stock	Final	V/μL
T4 DNA Ligase reaction buffer	10×	1×	8
P5 adapter and splint mix			9
P7 adapter and splint mix			9
ssDNA sample			9
PEG-8000	50%	20%	32
ddH_2_O			11
  f. Add 1 μL (30 U) T4 DNA Ligase and 1 μL T4 PNK (10 U) to the reaction mix. Carefully mix by pipetting up and down.
  g. Incubate ∼16 hours at 25°C in a thermocycler with the lid set to “off”.
  h. Incubate 10 minutes at 65°C with the lid set to 105°C to inactivate proteins.
  i. Use the Monarch Spin PCR & DNA Cleanup Kit with the standard cleanup protocol to clean up 80 μL of each sample. Change the ratio of sample: binding buffer to 3:1:
    i. Add 240 μL Buffer BZ to the solution. Mix well by pipetting up and down. Do not vortex.
    ii. Transfer 320 μL solution onto a spin column and spin for 1 minute at 16,000 g. Discard flow-through.
    iii. Add 200 μL Buffer WZ to the spin column and spin for 1 minute at 16,000 g. Discard flow-through.
    iv. Repeat step iv.
    v. Transfer the spin column to a fresh 1.5 mL sample tube. Spin empty for 1 minute at 16.000 g to remove residual buffer.
    vi. Transfer the spin column to a fresh 1.5 mL sample tube. Add 17 μL ddH_2_O to the center of the matrix to elute DNA. Spin for 1 minute at 16,000 g, then discard the spin column.
      ***Note:*** 2 μL of the final cleaned-up product can be used for analysis via Urea-PAGE gel (20% 19:1 acrylamide: bisacrylamide) or a TapeStation or Bioanalyzer. The adapters in this work are 33 nt and 34 nt long, meaning the expected ligated product is library length + 67 nt, in the case of N_30,_ 97 nt. If low yields are observed, refer to problem 3.
      **Pause point:** At this point, it is possible to store the samples at −20°C and continue the protocol at a later point.
4. Addition of index- and sequencing-primers via PCR.
  a. Prepare the following PCR mix:

**Table:** 

Reagent	Stock	Final	V/μL
adapter ligated DNA			15
NEBNext Ultra II Q5 Master Mix	2×	1×	25
Index primer (NEB primer set)			5
Universal primer (NEB primer set)			5
  b. Run the following PCR cycle:

**Table:** 

Steps	Temperature	Time	Cycles
Initial Denaturation	98°C	30 sec	1
Denaturation	98°C	10 sec	15 cycles
Annealing/Extension	65°C	75 sec	
Final extension	65°C	300 sec	1
Hold	4°C	forever
    **CRITICAL:** Do not use the same index primer for multiple samples! Make a note of which index primer is used for each sample and the correct index read of that primer. You will need to provide this information to the sequencer.
  c. Clean up using NucleoMag NGS Clean-up and Size Selection:
    i. Vortex NucleoMag solution until all beads are resuspended.
    ii. Add 45 μL bead solution to the finished PCR reaction, and incubate for 5 minutes.
    iii. Put on a magnetic rack, incubate for 5 minutes, and remove the supernatant.
    iv. Add 200 μL 80% Ethanol while on the magnetic rack, incubate 30 seconds, and remove supernatant.
    v. Repeat step iv. Ensure that as much liquid as possible is removed.
    vi. Air dry beads for a maximum of 5 minutes.
    vii. Remove from the magnetic rack. Add 33 μL of 0.1 TE buffer (10 mM Tris-HCl, pH 8.0, 1 mM EDTA), mix well by pipetting up and down to resuspend beads. Incubate 2 minutes.
    viii. Put on a magnetic rack, incubate 5 minutes.
    ix. Transfer 30 μL to a fresh 0.2 mL PCR tube.
  d. Confirm the success of the library preparation, e.g., using a TapeStation with a D1000 cassette if available.
    **Pause point:** At this point, it is possible to store the samples at −20°C and continue the protocol at a later point.
5. Based on the TapeStation concentration measurements, dilute and mix your library for loading concentrations.
  ***Note:*** For calculations, use the peak corresponding to the correct library length (for the same adapters and library length, this peak is at 154 bp), the peak corresponding to an adapter dimer (for the same adapters, this peak is at 124 bp) and the peak corresponding to a double insert (for the same adapters and library length, this peak is at 184 bp). Add these three peaks together to calculate the total sample library concentration.
  a. In low-binding tubes, dilute each sample library to 1 nM with 10 mM Tris, pH 8.5.
  b. Mix 10 μL of each 1 nM sample library in a low-binding tube. This yields a 1 nM mixed sample library.
  c. Prepare the final loading library by preparing this last dilution step and adding in the PhiX Control Library:

**Table:** 

Reagent	Stock	Final	V/μL
mixed sample library	1 nM	50 pM	5
PhiX Control V3	1 nM	50 pM	5
Tris pH 8.5	10 mM	10 mM	90
6. Prepare the iSeq 100 and sequencing cassette according to the manufacturer’s instructions:
  a. Load the sequencing cassette with the flow cell and library into the iSeq 100:
    i. Thaw the flow cell for 30 minutes at ∼25°C.
    ii. Unpack the thawed sequencing cassette.
    iii. Invert the sequencing cassette 5 times.
    iv. Tap the sequencing cassette on the table 5 times.
    v. Pierce foil protecting the library loading well with a pipette tip.
    vi. With a fresh pipette tip, load 20 μL of the final loading library into the library loading well.
    vii. Insert the flow cell into the sequencing cassette.
    viii. Insert the sequencing cassette into the iSeq 100.
  b. On the sequencer, set up a new run in Local Run Manager:
    i. Select “Generate FastQ” as the method.
    ii. Name your experiment.
    iii. Choose “Custom” as the library preparation protocol.
    iv. Choose 1 Index primer read (P7) with a read length of 6.
    v. Choose dual-read mode with a read length of 151 each.
    vi. In the sample table, add all the samples you prepared together with the corresponding Index read.
  c. In the sequencing software, click on sequencing. Follow the instructions on the sequencer, choose the new experiment created in the step above, and start the sequencer.

**Pause point:** At this point, it is possible to store the samples at −20°C and continue the protocol at a later point.

### Data analysis

**Timing: Days to weeks**

This final step provides a workflow for analyzing the sequencing data. First, we describe the general downstream processing, followed by multiple optional analysis modules for your specific data.

7. Quality control of sequencing itself using Illumina Sequencing Analysis Viewer.
  a. Download or transfer the entire folder containing sequencing results onto a machine with Illumina Sequencing Analysis Viewer installed.
  b. Open Illumina Sequencing Analysis Viewer. The software is available under the following link: https://support.illumina.com/sequencing/sequencing_software/sequencing_analysis_viewer_sav.html).
  c. At the top, click “Browse” and navigate to the folder containing the sequencing results. Open that folder.
  d. On the “Analysis” screen, many relevant metrics can be found, like quality statistics for reads per cycle, sequence, etc.
  e. Changing to “Imaging” and clicking the “Scatter Plot…” button allows plotting of different characteristics in an x/y scatter plot with each tile on the flow cell as a separate data point.
    i. Plot “% Occupied” on the x-axis.
    ii. Plot “% Pass Filter” on y-Axis.

***Note:*** If the flow cell was loaded correctly, you should see an approximately oval cloud of data points. If, instead, you see a diagonal line with x ≈ y, the flow cell was underloaded. A vertical band at % Occupied 95%–100% indicates overloading.

***Note:*** If the number of reads per sample is unexpectedly low, refer to problems 4 and 5.

8. Quality control of FASTQ files using FastQC.^15^
  **CRITICAL:** Quality control with FastQC should always be performed with raw FASTQ files, even after adapter trimming or filtering.
  a. If applicable, unzip the FASTQ files with gzip.
    >#!bin/bash
    >gunzip ∗.fastq.gz
  b. Install FastQC and MultiQC in a new environment using conda.
    >#!bin/bash
    >conda create -n fastqc-env fastqc multiqc -c bioconda -c conda-forge
  c. Activate the new environment and run FastQC.
    >#!bin/bash
    >conda activate fastqc-env
    >fastqc ∗.fastq
  d. FastQC generates.html outputs for each file it analyzes. You can either look at each of these individually or run MultiQC to explore them together.^16^
    >#!bin/bash
    >multiqc .
    ***Note:*** MultiQC uses any FASTQ output in the specified folder to generate its own output, which is simply a combined report of all the individual FastQC reports. We advise using MultiQC only to group analysis examples of the same processing stage together. For example, do not use it to group samples before and after adapter trimming, as these files may differ significantly and their combined report may not be useful.
9. Remove adapters using cutadapt.^17^
  a. Install cutadapt in a new environment using conda.
    >#!bin/bash
    >conda create -n cutadapt-env cutadapt -c bioconda -c conda-forge
  b. Activate the new environment and run cutadapt for trimming of paired-end reads.
    ***Note:*** This script assumes that the original FASTQ files are stored in the “../raw_data” folder with the naming convention “{var}_RX.fastq”, where X is either 1 or 2 (1 being the first read and 2 being the second read). Ensure that your data follows this format or adapt the script as required.
    ***Note:*** This script uses the P5 and P7 adapter sequences presented in this work for adapter trimming. Ensure that you change the above sequences to match those used when running the script.
    ***Note:*** The error tolerance for finding matching adapters is set to 0.2. This allows for a maximum of three mismatches when searching for adapters.
    >#!/bin/bash
    >source "$(conda info --base)/etc/profile.d/conda.sh"
    >
    ># Define an array of variable names
    >variables=("Ref" "Sup" "Pel")
    >
    ># Loop through each variable
    >for var in "${variables[@]}"; do
    > # Define input and output file names based on the variable
    > input_file_1="../raw_data/${var}_R1.fastq"
    > input_file_2="../raw_data/${var}_R2.fastq"
    > trimmed_output_file_1="${var}_trim_1.fastq"
    > trimmed_output_file_2="${var}_trim_2.fastq"
    >
    > # Run cutadapt to trim adapters
    > conda activate cutadapt-env
    > cutadapt -a "AGATCGGAAGAGCACACGTCTGAACTCCAGTCA" -A >"AGATCGGAAGAGCGTCGTGTAGGGAAAGAGTGT" --discard-untrimmed -o >"$trimmed_output_file_1" -p "$trimmed_output_file_2" >"$input_file_1" "$input_file_2"
    > conda deactivate
    > # Run FastQC on the trimmed output file
    > conda activate fastqc-env
    > fastqc "$trimmed_output_file_1"
    > fastqc "$trimmed_output_file_2"
    > conda deactivate
    >done
    >
    >echo "Processing complete for all variables."
  c. Confirm if adapter trimming was successful by looking at the FastQC output.
10. Filter sequences for correct length using seqkit.^18^
  a. Install seqkit using conda.
    >#!bin/bash
    >conda create -n seqkit-env seqkit -c bioconda -c conda-forge
  b. Activate the new environment and run the following script to filter your sequencing reads by length.
    >#!/bin/bash
    >source "$(conda info --base)/etc/profile.d/conda.sh"
    >
    ># Define an array of variable names
    >variables=("Ref" "Sup" "Pel")
    ># Loop through each variable
    >for var in "${variables[@]}"; do
    > # Define input and output file names based on the variable
    > input_file_1="../cut_adap/${var}_trim_1.fastq"
    > output_file_1="${var}_fil_1.fastq"
    > input_file_2="../cut_adap/${var}_trim_2.fastq"
    > output_file_2="${var}_fil_2.fastq"
    >
    > # Run seqkit to filter for length
    > conda activate seqkit-env
    > seqkit seq -m 30 -M 30 ${input_file_1} > ${output_file_1}
    > seqkit seq -m 30 -M 30 ${input_file_2} > ${output_file_2}
    > conda deactivate
    >
    > # Run FastQC on the filtered output file
    > conda activate fastqc-env
    > fastqc "$output_file_1"
    > fastqc "$output_file_2"
    > conda deactivate
    >done
    >
    >echo "Processing complete for all variables."
  c. Confirm if adapter trimming was successful by looking at the FastQC output.
    ***Note:*** All analysis modules are optional following this step and depend heavily on the samples you aim to analyze. All the scripts presented in this protocol are compatible with the data generated from sequencing the N_30_ library that was isolated above.
  d. Generate FASTA files from your FASTQ files for faster data processing.
    >#!bin/bash
    >seqkit fq2fa Ref_fil_1.fastq -o Ref_fil_1.fasta
    ***Note:*** It is not possible to use a wildcard character here, so you must process all files manually instead.
    **CRITICAL:** As our sequencing data originated from ssDNA only, we will now only process the R1 reads, which correspond to the original sequence in the droplets.
11. Calculate and plot nucleotide content in Python.^19^^,^^20^^,^^21^^,^^22^
  a. Install and import the relevant Python packages into a Python environment.
    >#!bin/bash
    >conda install pandas -c conda-forge
    >conda install matplotlib -c conda-forge
    >#!/usr/bin/env python3
    >import pandas as pd
    >import numpy as np
    >import matplotlib.pyplot as plt
  b. Use the following Python script to load the FASTA files into a pandas library in Python.
    >#!/usr/bin/env python3
    >
    >def read_fasta_to_dataframe(file_path):
    > sequences = []
    >
    > with open(file_path, 'r') as file:
    >  for i, line in enumerate(file):
    > if i % 2 == 1: # Every second line contains the sequence
    >  sequences.append(line.strip())
    >
    > # Create a DataFrame from the list of sequences
    > df = pd.DataFrame(sequences, columns=['Sequence'])
    >
    > return df
    >
    >variants = ["Ref", "Sup", "Pel"]
    >
    >#Create an empty dictionary
    >df_raw = {}
    >
    >#Store each dataframe generated by read_fasta_to_dataframe in df_raw under its variants name
    >for variant in variants:
    > file_path = f"../len_fil/{variant}_fil_1.fasta"
    > df_raw[variant] = read_fasta_to_dataframe(file_path)
  c. Calculate the nucleotide content per position for each sample.
    >#!/usr/bin/env python3
    >
    >def calculate_nucleotide_content(df):
    > # Get the maximum sequence length
    > max_length = df['Sequence'].str.len().max()
    >
    > # Initialize a dictionary to store nucleotide counts for each position
    > position_counts = {nucleotide: [0] ∗ max_length for nucleotide in 'AGCT'}
    >
    > # Count the nucleotides at each position
    > for sequence in df['Sequence']:
    >  for position, nucleotide in enumerate(sequence):
    >  if nucleotide in position_counts:
    >  position_counts[nucleotide][position] += 1
    >
    > # Convert counts to percentages
    > position_percentages = {nucleotide: [] for nucleotide in 'AGCT'}
    > for position in range(max_length):
    >  total_count = sum(position_counts[nuc][position] for nuc in 'AGCT')
    >  if total_count > 0:
    >   for nucleotide in 'AGCT':
    >   position_percentages[nucleotide].append((position_counts[nucleotide][position] / total_count) ∗ 100)
    >  else:
    >   for nucleotide in 'AGCT':
    >   position_percentages[nucleotide].append(0)
    >
    > # Create a DataFrame from the position percentages
    > percentage_df = pd.DataFrame(position_percentages)
    >
    > return percentage_df
    >
    >#Create an empty dictionary
    >df_perc = {}
    >
    >#Store each dataframe generated by calculate_nucleotide_content in df_perc under its variants name
    >for variant in variants:
    > df_perc[variant] = >calculate_nucleotide_content(df_raw[variant])
  d. Plotting the nucleotide content per position for each sample in an individual plot.
    >#!/usr/bin/env python3
    >
    >#Some generic settings to match the asthetics of this paper
    >plt.rcParams['font.family'] = 'Arial'
    >plt.rcParams['font.size'] = 9
    >plt.rcParams['font.weight'] = 'bold'
    >
    >for variant, perc in df_perc.items():
    > #Plots the lines for G, A, C and T
    > plt.figure(figsize=(2.5, 2))
    > plt.plot(perc.index + 1, perc["G"], label="G", color="orange")
    > plt.plot(perc.index + 1, perc["A"], label="A", color="red")
    > plt.plot(perc.index + 1, perc["C"], label="C", color="blue")
    > plt.plot(perc.index + 1, perc["T"], label="T", color="green")
    >
    > #Labels and design
    > plt.grid()
    > plt.title(f"{variant} Content", weight="bold", fontsize=10)
    > plt.xlabel("#Base", weight="bold", fontsize=10)
    > plt.ylabel("Base content / %", weight="bold", fontsize=10)
    > plt.yticks(np.arange(15, 40, 5), fontsize=9, weight='bold')
    >
    > #Save each figure individually
    > plt.savefig(f"{variant}_content.pdf", bbox_inches="tight", pad_inches=0.1)
    > plt.show()
  e. Creating the corresponding legend as a separate file.
    >#!/usr/bin/env python3
    >
    >#Creating the legend as a seperate file
    >plt.figure(figsize=(4, 1))
    >handles = [
    > plt.Line2D([0], [0], color='orange', lw=4),
    > plt.Line2D([0], [0], color='red', lw=4),
    > plt.Line2D([0], [0], color='blue', lw=4),
    > plt.Line2D([0], [0], color='green', lw=4),
    >]
    >labels = ['G', 'A', 'C', 'T']
    >plt.legend(handles, labels, loc='center', fontsize=9, ncol=4)
    >
    ># Turn off axes and save legend figure
    >plt.axis('off')
    >plt.savefig("legend_content.pdf", bbox_inches="tight")
  f. Calculating the difference between each sample and the “Reference” sample to see the change in distribution before and after samples were taken.
    >#!/usr/bin/env python3
    >
    >#Creating an empty dictionary
    >df_diff = {}
    >
    >#Subtracting the Reference from each sample
    >for variant in variants:
    > df_diff[variant] = df_perc[variant] - df_perc["Ref"]
  g. Plotting the new difference once again in individual plots for each sample.
    >#!/usr/bin/env python3
    >
    >#Plotting the df_diff for each sample.
    >for variant, diff in df_diff.items():
    > plt.figure(figsize=(2.5, 2))
    > plt.plot(diff.index + 1, diff["G"], label="G", color="orange")
    > plt.plot(diff.index + 1, diff["A"], label="A", color="red")
    > plt.plot(diff.index + 1, diff["C"], label="C", color="blue")
    > plt.plot(diff.index + 1, diff["T"], label="T", color="green")
    > plt.grid()
    > plt.title(f"{variant} Difference", weight="bold", fontsize=10)
    > plt.xlabel("#Base", weight="bold", fontsize=10)
    > plt.ylabel(r"$∖Delta$ Base content / %", weight="bold", fontsize=10)
    > plt.yticks(np.arange(-8, 9, 4), fontsize=9, weight='bold')
    >
    > plt.savefig(f"{variant}_difference.pdf", bbox_inches="tight", pad_inches=0.1)
12. Calculating folding and minimum-free energy values.
  a. Install Vienna RNA package per suppliers’ instructions^23^^,^^24^^,^^25^: https://www.tbi.univie.ac.at/RNA/documentation.html#install.
  b. Use the following script to calculate the minimum free energy structure using RNAfold, then extract the minimum free energy from the result file.
    ***Note:*** The flag ‘-P DNA’ flag is parsed, so DNA folding parameters are used instead of RNA parameters. The parameters used by RNAfold are from Matthews et al.^26^
    ***Note:*** The script produces four output files: the regular RNAfold output, named {var}_rnafold.txt; a file containing the dot-bracket structure and minimum free energy values, named {var}_deltaG.txt; and files containing either just the dot-bracket structure or just the energy values, named {var}_dot_bracket.txt and {var}_energy.txt respectively.
    >#!/bin/bash
    >
    >file_list="file_list.txt"
    >rnafold_output_dir="rnafold_output"
    >mkdir -p $rnafold_output_dir
    >
    >while IFS= read -r input_fasta; do
    > filename=$(basename "$input_fasta")
    > rnafold_output="$rnafold_output_dir/${filename%.fasta}_rnafold.txt"
    > deltaG_output="$rnafold_output_dir/${filename%.fasta}_deltaG.txt"
    > RNAfold -i "$input_fasta" --noPS --noconv -P DNA > "$rnafold_output"
    > grep "(" "$rnafold_output" > "$deltaG_output"
    > echo "Processed RNAfold and extracted deltaG values for $output_fasta -> $rnafold_output"
    >
    > # Python processing block
    > python3 <<EOF
    ># Define file paths directly from bash variables
    >deltaG_file = "${deltaG_output}"
    >dot_bracket_file = "./${rnafold_output_dir}/${filename%.fasta}_dot_bracket.txt"
    >energy_file = "./${rnafold_output_dir}/${filename%.fasta}_energy.txt"
    >
    ># Read input file and process line by line
    >with open(deltaG_file, 'r') as infile, ∖
    > open(dot_bracket_file, 'w') as dot_out, ∖
    > open(energy_file, 'w') as energy_out:
    > for line in infile:
    >  # Extract first 60 characters (dot-bracket notation)
    >  dot_bracket = line[:30].strip()
    >  dot_out.write(dot_bracket + '∖n')
    >
    >  # Extract energy value starting from character 61 onward,
    >  # stripping spaces and brackets (retaining only numeric parts)
    >  energy_value_raw = line[31:] # Start reading from character 61 onward
    >  energy_value = ''.join(c for c in energy_value_raw if c.isdigit() or c == '.' or c == '-')
    >  energy_out.write(energy_value + '∖n')
    >print(f"Generated {dot_bracket_file} and {energy_file}")
    >EOF
    done < "$file_list"
  c. In a Python environment, import the relevant Python packages.
    >#!/usr/bin/env python3
    >import pandas as pd
    >import matplotlib.pyplot as plt
    >import numpy as np
  d. Load the energy values for each sample from {var}_fil_1_energy.txt.
    >#!/usr/bin/env python3
    >
    >file_list = ["Ref", "Sup", "Pel"]
    >df = {}
    >
    >for file in file_list:
    > df[file] = >pd.read_csv(f"rnafold_output/{file}_fil_1_energy.txt", header=None)
    > df[file].rename(columns={0:"values"}, inplace=True)
  e. Sort the values into bins, count them, and calculate the frequency of each bin with the following script.
    >#!/usr/bin/env python3
    >
    >num_bins = 10
    >min_value = -10
    >max_value = 0
    >bin_edges = np.linspace(min_value, max_value, num_bins + 1)
    >x_values = np.linspace(min_value, max_value, num_bins + 2)
    >
    >df_counts = {}
    >df_freq = {}
    >
    >for file in file_list:
    > df[file]["bin"] = np.digitize(df[file]["values"], bin_edges)
    > df_counts[file] = np.bincount(df[file]["bin"])
    > df_freq[file] = df_counts[file] / len(df[file])
  f. Plot the MFE frequencies as a distribution plot.
    >#!/usr/bin/env python3
    >
    >plt.rcParams['font.family'] = 'Arial'
    >plt.rcParams['font.size'] = 9
    >plt.rcParams['font.weight'] = 'bold'
    >
    >plt.figure(figsize=(2.5, 2.5))
    >
    >for file in file_list:
    > plt.plot(x_values, df_freq[file], label = file)
    >
    >plt.legend(loc="upper left")
    >plt.xlabel(r"Minimum free energy", weight="bold", fontsize=10)
    >plt.ylabel("relative occurence", weight="bold", fontsize=10)
    >
    >plt.savefig(fname="deltaG.pdf", bbox_inches='tight', pad_inches=0.1)
    >plt.show()
  g. Calculate the average MFE values per sample.
    >#!/usr/bin/env python3
    >
    >df_avg = {}
    >
    >for file in file_list:
    > df_avg[file] = df[file]["values"].mean()
    > print(f"{file}: {round(float(df_avg[file]), 3)}")
13. Discovering enriched motifs in samples compared to a control sample.
  a. Install the meme-suite per suppliers’ instructions^27^^,^^28^: https://meme-suite.org/meme/doc/install.html.
  b. Use streme from the meme suite to find enriched motifs in a sample compared to a reference.
    **CRITICAL:** It is essential to run this with the --rna flag! Using a DNA library instead will automatically force the algorithm to generate and search the reverse complement of each sequence, which was not present in the original experiment. This will result in false positives that are the reverse complement of real motifs.
    ***Note:*** Adjusting the maximum length of motifs to be discovered can drastically increase or decrease the number found. For example, we found a total of ten statistically significant motifs with a maximum motif width (*maxw*) of 20; however, with a *maxw* of 10, we found over twenty. This is due to the way the algorithm works: it deletes parts of sequences that carry a motif so that many variations of the same motif are not found at the same sites.
    ***Note:*** This algorithm is very computationally taxing as it was designed for far fewer, longer reads, and it is incapable of multi-threading. For a sample of 370,000 reads and a reference of 1,500,000 reads, we found that it took approximately one day on an i9 14900k processor and required approximately 25 GB of RAM.
    >#!/usr/bin/env python3
    >streme –p ../len_fil/pel_fil_1.fasta –o streme_output –n ../len_fil/sup_fil_1.fasta –maxw 10 --rna
  c. From the output files, you can get the Motif information from the streme.txt file. Copy these (starting with the line “MOTIF 1…” and paste them into a new file called motifs.txt.
  d. Import all relevant libraries in Python. Install all that you are missing analogues to the above explanation (Step 11A).^29^
    >#!/usr/bin/env python3
    >
    >import numpy as np
    >import logomaker
    >import pandas as pd
    >import matplotlib.pyplot as plt
    >import re
    >from collections import defaultdict
  e. Use the following script to open both motifs.txt as well as sites.tsv for plotting of logos and positional information of logos.
    >#!/usr/bin/env python3
    >
    ># Load the text file
    >with open("motifs.txt", "r") as f:
    > raw_data = f.read()
    >
    ># Regex to split motifs
    >motif_blocks = re.findall(r'(MOTIF∖s+∖d+.∗?)(?=MOTIF∖s+∖d+|∖Z)', raw_data, re.DOTALL)
    >
    >motifs = []
    >
    ># Map index to base
    >base_order = ['A', 'C', 'G', 'T']
    >
    >for block in motif_blocks:
    > # Extract lines with matrix values only
    > lines = block.strip().splitlines()
    > matrix_lines = [line for line in lines if re.match(r'ˆ∖s∗∖d∗∖.?∖d+∖s+', line)]
    >
    > motif = defaultdict(list)
    >
    > for line in matrix_lines:
    >  probs = list(map(float, line.strip().split()))
    >  for i, base in enumerate(base_order):
    >  motif[base].append(probs[i])
    >
    > motifs.append(dict(motif))
    >
    >df = pd.read_csv("sites.tsv", sep="∖t")
  f. Calculate information content to plot logos with information content on the y-axis instead of nucleotide content (column 1 of final figure).
    >#!/usr/bin/env python3
    >
    >def calculate_information_content(motif):
    > ic = np.log2(4) + (motif ∗ np.log2(motif)).sum(axis=1)
    > ic = ic.fillna(0) # Handle NaNs resulting from log2(0)
    > return ic
  g. Calculate positional hits for each motif for later plotting (column 3 of final figure).
    >#!/usr/bin/env python3
    >
    >position_counts = defaultdict(lambda: defaultdict(int))
    >total_counts = defaultdict(int)
    >
    >for _, row in df.iterrows():
    > # Skip rows with missing start/end
    > if pd.isna(row['site_Start']) or pd.isna(row['site_End']):
    >  continue
    >
    > motif = row['motif_ALT_ID']
    > try:
    >  start = int(row['site_Start'])
    >  end = int(row['site_End'])
    > except ValueError:
    >  continue # skip if conversion fails
    >
    > # Only count positions within the 1–30 range
    > for pos in range(start, end + 1):
    >  if 1 <= pos <= 30:
    >  position_counts[motif][pos] += 1
    >
    > total_counts[motif] += 1
  h. Build a dataframe storing all information for column 3 (positional hits of motifs).
    >#!/usr/bin/env python3
    >
    ># Create list of all unique motifs
    >motifs_ids = list(position_counts.keys())
    >
    ># Build table for column 1-3
    >data = []
    >
    >for motif in motifs_ids:
    > row = {'motif_ALT_ID': motif}
    > total = total_counts[motif]
    > row['total'] = total
    >
    > # Fill in positions 1–30
    > for pos in range(1, 31):
    >  row[f'pos{pos}'] = position_counts[motif].get(pos, 0)
    >
    > data.append(row)
    >
    ># Convert to DataFrame
    >result_df = pd.DataFrame(data)
  i. Define a function for numerical sorting of indexes (needed to correctly sort dataframes).
    >#!/usr/bin/env python3
    >
    >def numeric_sort_index(df):
    > # Extract the number from strings like "STREME-11"
    > nums = >df.index.to_series().str.extract(r'(∖d+)$').astype(int)[0]
    > return df.iloc[nums.argsort()]
  j. Build a dataframe for column 4 (starting position of motifs).
    >#!/usr/bin/env python3
    >
    ># Build df_start used for column 4
    >df_start = (
    > df.dropna(subset=['site_Start'])
    >  .assign(site_Start=lambda x: x['site_Start'].astype(int))
    >  .query('1 <= site_Start <= 30')
    >  .groupby(['motif_ALT_ID', 'site_Start'])
    >  .size()
    >  .unstack(fill_value=0)
    >)
    >
    ># Ensure all positions exist
    >for pos in range(1, 31):
    > if pos not in df_start.columns:
    > df_start[pos] = 0
    >df_start = df_start[sorted(df_start.columns)]
    >
    ># Sort index numerically
    >df_start = numeric_sort_index(df_start)
  k. Build a dataframe for column 5 (end sites of motifs).
    >#!/usr/bin/env python3
    >
    ># Build df_end used for column 5
    >df_end = (
    > df.dropna(subset=['site_End'])
    >  .assign(site_End=lambda x: x['site_End'].astype(int))
    >  .query('1 <= site_End <= 30')
    >  .groupby(['motif_ALT_ID', 'site_End'])
    >  .size()
    >  .unstack(fill_value=0)
    >)
    >
    >for pos in range(1, 31):
    > if pos not in df_end.columns:
    >  df_end[pos] = 0
    >df_end = df_end[sorted(df_end.columns)]
    >df_end = numeric_sort_index(df_end)
  l. Calculate fractions of hits from the three dataframes generated above.
    >#!/usr/bin/env python3
    >
    ># Fractions of hits at each position used for column 3
    >result_fraction_df = result_df.set_index('motif_ALT_ID').copy()
    >for pos in range(1, 31):
    > result_fraction_df[f'pos{pos}'] = (
    >  result_fraction_df[f'pos{pos}'] / >result_fraction_df['total'].replace(0, np.nan)
    > ).fillna(0)
    >
    ># Sort same as df_start/df_end
    >result_fraction_df = numeric_sort_index(result_fraction_df)
    >
    >#Fractions of start and end used for column 4 and 5
    >
    >df_start_frac = df_start.div(df_start.sum(axis=1), >axis=0).fillna(0)
    >df_end_frac = df_end.div(df_end.sum(axis=1), axis=0).fillna(0)
  m. Generate a final dataframe that will store all data used for plotting.
    >#!/usr/bin/env python3
    >plot_data = []
    >
    ># Loop through motifs in the already-sorted result_df
    >for mid in result_df['motif_ALT_ID']:
    > plot_data.append({
    >  'id': mid,
    >  'logo_matrix': >pd.DataFrame(motifs[result_df['motif_ALT_ID'].tolist().index(mid)]),
    >  'pos_fraction': result_fraction_df.loc[mid, [f'pos{p}' >for p in range(1, 31)]].values,
    >  'start_fraction': df_start_frac.loc[mid].values,
    >  'end_fraction': df_end_frac.loc[mid].values
    > })
  n. Plot a master figure with five columns per motif, with the first column being the logo with information content as the y-axis, the second column logo with nucleotide content as the y-axis, the third column showing positional hits (frequency that logo appears at each site), the fourth column showing starting sites of logos, and the fifth column showing ending positions of logos.
    >#!/usr/bin/env python3
    >
    ># Number of motifs
    >num_motifs = len(plot_data)
    >
    ># Create subplots
    >fig, axes = plt.subplots(num_motifs, 5, figsize=(5 ∗ 35 / 25.4, 18 / 25.4 ∗ num_motifs))
    >plt.subplots_adjust(wspace=0.5, hspace=0.7)
    >
    ># Define the titles for each column
    >col_titles = [
    > "Logo inf",
    > "Logo nuc",
    > "Motif pos",
    > "Motif start",
    > "Motif end"
    >]
    >
    ># Position titles above the top row's axes
    >for col_idx, title in enumerate(col_titles):
    > ax = axes[0][col_idx]
    > ax.set_title(title, fontsize=12, fontweight='bold', pad=20)
    >
    ># Loop over motifs
    >for i, motif_entry in enumerate(plot_data):
    > row_axes = axes[i] # 5 axes in a row
    > motif_name = motif_entry['id']
    >
    > # Extract precomputed data
    > df_logo = motif_entry['logo_matrix']
    > pos_fraction = motif_entry['pos_fraction']
    > start_fraction = motif_entry['start_fraction']
    > end_fraction = motif_entry['end_fraction']
    >
    > # Calculate information content
    > ic = calculate_information_content(df_logo)
    > df_ic = df_logo.mul(ic, axis=0)
    >
    > color_scheme = {
    >  'A': 'red',
    >  'C': 'orange',
    >  'G': 'blue',
    >  'T': 'green'
    > }
    > # Column 1: Information content logo
    > logomaker.Logo(df_ic, ax=row_axes[0], color_scheme=color_scheme)
    > row_axes[0].set_xticks([])
    > row_axes[0].set_ylim(0, 2)
    >
    > # Column 2: Nucleotide content logo
    > logomaker.Logo(df_logo, ax=row_axes[1], color_scheme=color_scheme)
    > row_axes[1].set_xticks([])
    > row_axes[1].set_ylim(0, 1)
    >
    > # Column 3: Position hits
    > row_axes[2].plot(range(1, 31), pos_fraction, color='black')
    > row_axes[2].set_ylim(0, 1)
    > row_axes[2].set_xlim(1, 30)
    > row_axes[2].set_xticks([])
    >
    > # Column 4: Start positions
    > row_axes[3].plot(range(1, 31), start_fraction, color='black')
    > row_axes[3].set_ylim(0, 0.1)
    > row_axes[3].set_xlim(1, 30)
    > row_axes[3].set_xticks([])
    >
    > # Column 5: End positions
    > row_axes[4].plot(range(1, 31), end_fraction, color='black')
    > row_axes[4].set_ylim(0, 0.1)
    > row_axes[4].set_xlim(1, 30)
    > row_axes[4].set_xticks([])
    >
    > # Y-axis styling
    > for ax in row_axes:
    >  ax.tick_params(axis='y', labelsize=8)
    >  for tick in ax.get_yticklabels():
    >  tick.set_fontsize(8)
    >  tick.set_fontweight('bold')
    >
    ># Save & show
    >plt.savefig("combined_motifs_all_in_one.pdf", bbox_inches='tight')
    >plt.show()
14. Calculate the abstracted structure of sequences and analyze them for enrichment of specific motifs.
  a. Install RNAshapes in a new environment using conda:^30^
    >#!bin/bash
    >conda create -n RNAshapes-env RNAshapes -c bioconda
  b. Run the following script to generate the abstracted structure for all samples.
    >#!/bin/bash
    >source "$(conda info --base)/etc/profile.d/conda.sh"
    >
    >variables=("Ref" "Sup" "Pel")
    >
    >conda activate RNAshapes-env
    >for var in "${variables[@]}"; do
    > RNAshapes -c 0 < ../len_fil/${var}_fil_1.fasta > >${var}_abstract.txt
    > echo "${var} finished computing"
    >
    >done
    >conda deactivate
    >
    >echo "Finished the script"
  c. Import all relevant libraries in Python. Install all that you are missing analogues to the above instructions (Step 11A).
    >#!/usr/bin/env python3
    >
    >import numpy as np
    >import pandas as pd
    >import matplotlib.pyplot as plt
  d. Use the following script to load the data into a Python dataframe.
    >#!/usr/bin/env python3
    >
    >def RNAshaper_to_dataframe(file_path):
    > energies = []
    > dotbrackets = []
    > abstracted_dotbrackets = []
    >
    > with open(file_path, 'r') as file:
    >  lines = file.readlines()
    >  total_lines = len(lines)
    >
    >  # Loop through the file and look for header lines (those starting with '>')
    >  for i in range(total_lines):
    >    if lines[i].startswith('>'):
    >    # Attempt to get the third line after the header (i+2)
    >    if i + 2 < total_lines:
    >     line = lines[i + 2].strip()
    >     components = line.split()
    >
    >     # Make sure the line has exactly 3 components
    >     if len(components) == 3:
    >      try:
    >       energy = float(components[0])
    >       dotbracket = components[1]
    >       abstracted_dotbracket = components[2]
    >
    >       energies.append(energy)
    >       dotbrackets.append(dotbracket)
    >       abstracted_dotbrackets.append(abstracted_dotbracket)
    >      except ValueError:
    >       # Skip lines where energy is not a float
    >       continue
    >
    > df = pd.DataFrame({
    >  'Energy': energies,
    >  'DotBracket': dotbrackets,
    >  'Abstracted': abstracted_dotbrackets
    > })
    >
    > return df
    >#!/usr/bin/env python3
    >
    >variables = ("Ref", "Sup", "Pel")
    >
    >df = {}
    >
    >for var in variables:
    > file_path = f"{var}_abstract.txt"
    > df[var] = RNAshaper_to_dataframe(file_path)
  e. Count the occurrences of each abstracted structure.
    >#!/usr/bin/env python3
    >
    >def count_abstracted_structures(df):
    > # Count occurrences of each unique structure in the 'Abstracted' column
    > structure_counts = df['Abstracted'].value_counts()
    >
    > # Create a new DataFrame with the structure and its count
    > result_df = pd.DataFrame({
    >  'Structure': structure_counts.index,
    >  'Occurrences': structure_counts.values
    > })
    >
    > return result_df
    >
    >df_abs = {}
    >
    >for var in variables:
    > df_abs[var] = count_abstracted_structures(df[var])
  f. Plot the occurrences of different structures in a grouped bar graph.
    >#!/usr/bin/env python3
    >
    ># Dynamically collect all unique "Structure" values across all df_abs DataFrames
    >all_categories = sorted(
    > set().union(∗[df_abs[var]["Structure"] for var in variables]),
    > key=lambda x: (len(x), x) # First sort by length, then alphabetically
    >)
    >
    ># Reindex all DataFrames in df_abs to include all categories, filling missing ones with 0
    >for var in variables:
    > df_abs[var] = >df_abs[var].set_index("Structure").reindex(all_categories, fill_value=0).reset_index()
    >
    >bar_width = 0.8 / len(variables) # Adjust bar width based on the number of variables
    >x = np.arange(len(all_categories))
    >
    >plt.figure(figsize=(2.5, 2))
    >
    ># Loop through variables and plot each one
    >for i, var in enumerate(variables):
    > plt.bar(
    >  x + (i - len(variables) / 2) ∗ bar_width, # Shift bars >for each variable
    >  df_abs[var]["Occurrences"] / len(df[var]) ∗ 100, # Normalize occurrences by total sequences
    >  width=bar_width,
    >  label=var.capitalize() # Use variable names as labels
    > )
    >
    >plt.xlabel("Abstracted Structures", size=10, fontweight="bold")
    >plt.ylabel("Percentage of Sequences", size=10, fontweight="bold")
    >plt.xticks(x, all_categories, rotation=45)
    >plt.yscale("log")
    >
    >plt.legend()
    >
    >plt.savefig("Abstracted_Structures.pdf", bbox_inches="tight", pad_inches=0.1)
    >plt.show()
  g. Calculate the average stem length per sample and write it in a new dataframe.
    >#!/usr/bin/env python3
    >
    >def count_stem_length(df):
    > # Calculate the count of '(' for each entry in the 'DotBracket' column
    > df['StemLength'] = df['DotBracket'].apply(lambda x: x.count('('))
    > return df
    >
    >for var in variables:
    > count_stem_length(df[var])
    >
    >def count_stem_length_occurences(df):
    > # Count occurrences of each unique structure in the 'Abstracted' column
    > structure_counts = df['StemLength'].value_counts()
    >
    > # Create a new DataFrame with the structure and its count
    > result_df = pd.DataFrame({
    >  'Length': structure_counts.index,
    >  'Occurrences': structure_counts.values
    > })
    >
    > return result_df
    >
    >df_stem = {}
    >
    >for var in variables:
    > df_stem[var] = >count_stem_length(df[var]).sort_values("Length")
  h. Plot the density of stem length per sample in a density plot.
    >#!/usr/bin/env python3
    >
    ># Calculate max_length dynamically from all df_stem DataFrames
    >max_length = max([df_stem[var]["Length"].max() for var in variables])
    >
    >plt.figure(figsize=(2.5, 2.5))
    >
    ># Loop through each variable to plot its density
    >for var, color in zip(variables, ["purple", "blue", "green", "orange", "red", "cyan", "brown"]): # Add more colors as needed
    > # Normalize occurrences by total sequences and plot the line
    > plt.plot(
    >  df_stem[var]["Length"],
    >  df_stem[var]["Occurrences"] / len(df[var]) ∗ 100,
    >  alpha=0.7,
    >  label=var.capitalize(),
    >  color=color
    > )
    > # Fill under the curve for better visualization
    > plt.fill_between(
    >  df_stem[var]["Length"],
    >  df_stem[var]["Occurrences"] / len(df[var]) ∗ 100,
    >  alpha=0.3,
    >  color=color
    > )
    >
    ># Labels and other configurations
    >plt.xlabel("Stem Length", size=10, fontweight="bold")
    >plt.ylabel("Percentage of Sequences", size=10, fontweight="bold")
    >plt.xticks(np.arange(0, max_length + 1, 2)) # Dynamically adjust x-ticks based on max_length
    >plt.legend()
    >
    ># Save and show plot
    >plt.savefig("StemLength_Density.pdf", bbox_inches="tight", pad_inches=0.1)
    >plt.show()

## Expected outcomes

**Figure 1:**
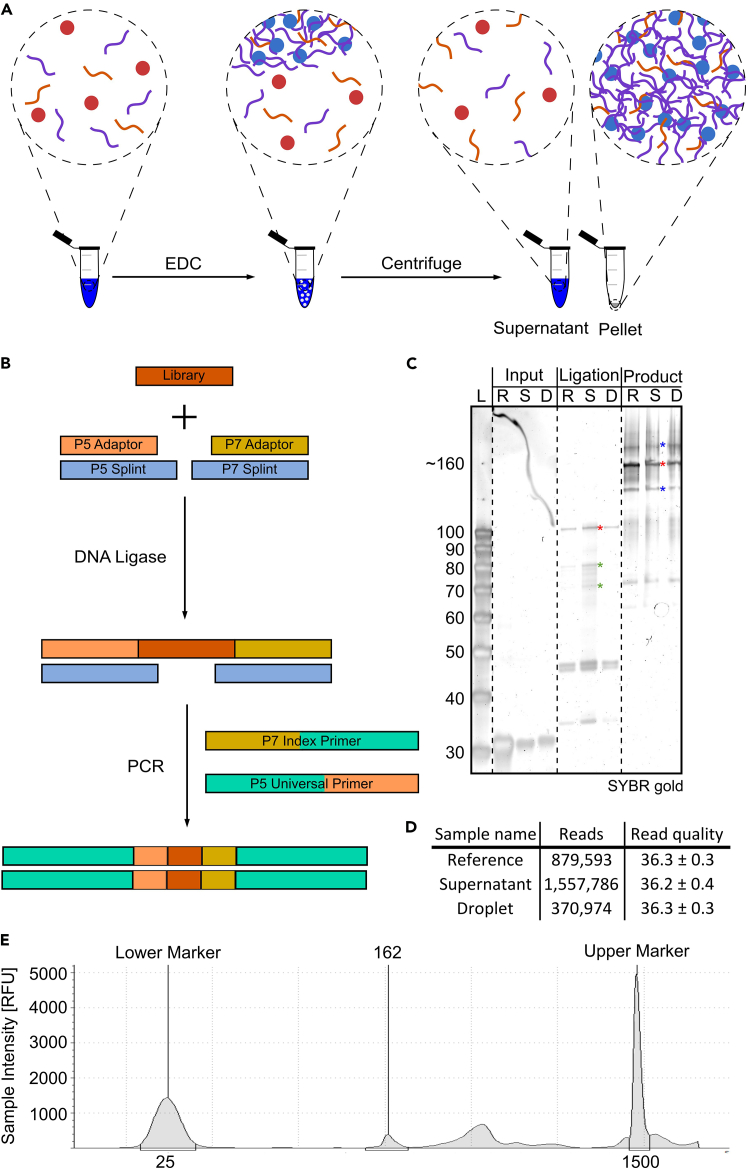
ssDNA isolation and library preparation (A) Prior to the addition of fuel, the peptide, polyU RNA and ssDNA molecules are mixed in solution, and do not form supramolecular assemblies. When fuel (EDC) is added, phase separation occurs, forming coacervate droplets that sequester the ssDNA molecules from the polymer-poor phase. The phases are separated via centrifugation to yield the polymer-poor and polymer-rich phases, from which the ssDNA populations are isolated. For a complete characterization of this droplet system, please refer to Donau et al., 2020.^2^ (B) Library preparation relies on the orientation-specific ligation of two adapter molecules to the recovered ssDNA oligonucleotides. After the ligation, the sequencing primers are added in a PCR using primers with overhangs. (C) Urea-PAGE is performed following library preparation. R = Reference, S = Supernatant, D = Droplets. ‘Input’ corresponds to the samples obtained from the droplets, while the ‘R’ sample is the naïve input library. ‘Ligation’ corresponds to the product of the first step in B and shows a clear band (red star) corresponding to the expected full-length ligation product, as well as a weaker smear (green stars) corresponding to the two partially adapter-ligated products. Bands at 45 nt correspond to the adapters and splint, and bands at ∼30 correspond to unreacted input ssDNA. ‘Product’ shows the full-length sequencing library, with the correct PCR band being marked with a red star. Several additional bands are visible, including two prominent bands (blue stars), which are located approximately 30 nt above and below the main PCR band. These correspond to the no-insert (adapter dimer) and double-insert ligation products. The smear and bands below correspond to indexing primers. (E) Electropherogram of a TapeStation run of a prepared library. Only the peak corresponding to the correct library size (∼160 bp) is used to calculate the correct library dilution for loading onto the sequencer.

The results of successful isolation of ssDNA from droplets (Figure 1A) and library preparation (Figure 1B) can be verified on a urea-PAGE gel. The ssDNA isolated from complex coacervates should produce a distinct band (Figure 1C, lane 4). Similarly, the ligation reaction (Figure 1C, lanes 5–7) and the final PCR (Figure 1C, lanes 8–10) should produce a prominent product band (marked with a red star). Note that bands corresponding to the adapter dimers (no insert) and double-insert products (concatemers) are expected (Figure 1C, lanes 8–10). These consume only a small proportion of the reads in the sequencing data and can therefore be disregarded, given that their yield is significantly lower compared to the target library (Figure 1C, lanes 8–10). These reads will be removed during the filtering of sequencing results downstream. Using the library shown here with a loading concentration of 12 pM, we obtained 2.8 million reads across three samples (Figure 1D). This comparably low yield is likely due to the high PhiX spike-in concentration (50 pM), but it still provides high-quality data with sufficient depth for robust statistics.

**Figure 2:**
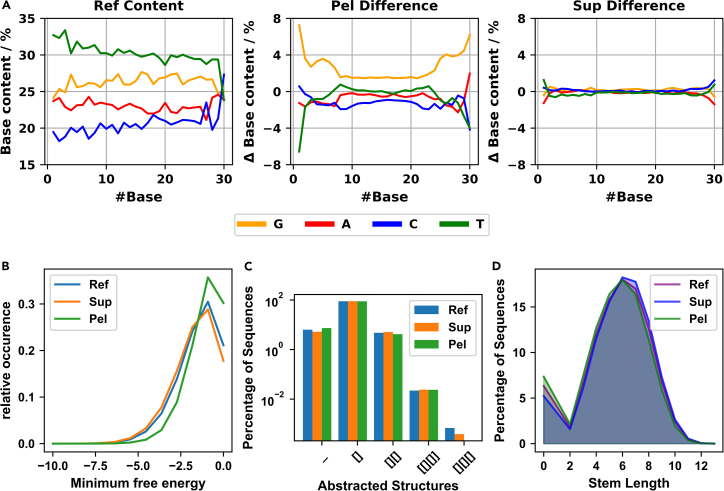
Results generated during data analysis (A) The base composition of the Reference sample enables the identification of biases in the naive library; most of these result from solid-phase synthesis (left panel). Difference in base composition between the samples from the polymer-rich phase (Pel = Droplet, middle panel) and the polymer-poor phase (Sup = Supernatant, right panel). (B) Distribution of minimum free energies of folding for sequencing reads of each sample. (C) Occurrence of different abstracted structures in each sample. [] represents a single stem, [][] two stems, [[][]] is a stem containing two secondary side stems, and [][][] represents three separate stems. (D) Distribution of intramolecular stem lengths in each sample.

**Figure 3:**
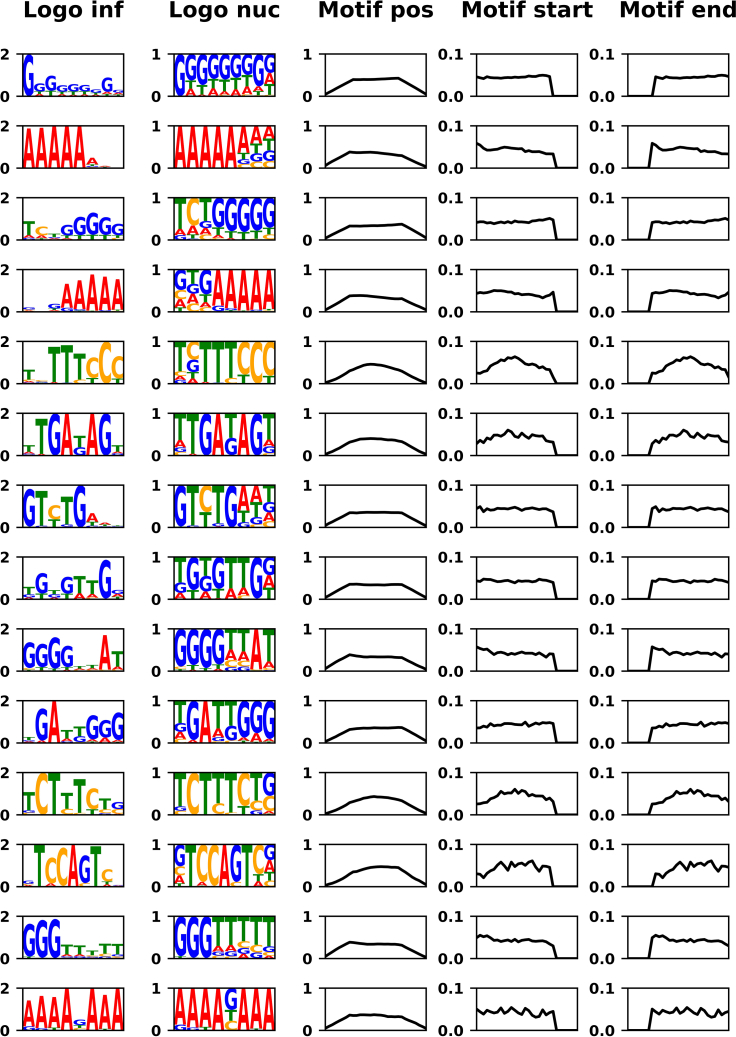
List of all statistically significant motifs identified with STREME “Logo inf” shows sequence logos scaled by information content (positions with higher information content appear larger). “Logo nuc” shows sequence logos based only on nucleotide frequencies. The columns “Motif start” and “Motif end” report the counts of motif start and end positions, respectively.

Data analysis should produce results similar to those in Figure 2. These include base composition (Figure 2A), difference in minimum free energy of folding (Figure 2B), abstract secondary structure classes (Figure 2C), and average intramolecular hybridization length (Figure 2D). We would like to emphasize motif discovery using STREME,^27^^,^^28^ which identifies enriched sequence motifs. Using an e-value cutoff of ≤0.05, we identified a total of 14 statistically significant motifs in our data (Figure 3).

We encourage researchers to analyze their dataset further to identify system-specific results. In our associated research manuscript, the results of the base-composition analysis prompted targeted analyses of reads starting or ending in guanosines (see Figure S6 in the associated research manuscript), while the motif discovery analysis informed searches for runs of G or A in droplet samples (see Figure 2F in the associated research manuscript). Follow-up experiments in the droplet system verified specific interactions between ssDNA sequences and RNA or peptide droplet components.

## Limitations

This protocol is optimized for transient complex coacervates. As implementations of protocell-like compartments vary widely between, the recovery steps are system-specific and may differ in static droplet systems or vesicle-based models, for example. While these models can reuse the library preparation and analysis modules, alternative ssDNA extraction methods that are compatible with the system matrix must be developed. If RNA rather than ssDNA is used as the nucleic acid component, both recovery and library preparation require re-optimization.

Our analysis pipeline is modular but not exhaustive. It provides a baseline of base composition, predicted folding energies, and motif discovery for short oligonucleotides. Researchers with different systems (e.g., longer sequences or double-stranded sequences, or RNA) may need to extend or develop additional modules to meet their specific analysis needs.

## Troubleshooting

### Problem 1

The droplet solution does not turn turbid upon the addition of EDC (Step 1C).

### Potential solution

• Make sure to use a fresh solution of dry EDC, as the compound is not stable in the presence of water. If the powder forms clumps, it may be too wet, and a new, pristine stock should be opened. For this reason, EDC should always be stored in completely dry conditions.
• Confirm the identity and pH of the peptide stock. Impurities can result in poor or no droplet formation or cause the peptide to precipitate, particularly at low pH values. Always store at pH 5.2 at 4°C.
• High concentrations of salts (e.g., in buffers or peptide solution due to multiple pH adjustments) can also inhibit droplet formation.
• Improperly stored polyU RNA will hydrolyze over time, which leads to inhibition of droplet formation. Make sure that the polyU RNA is always stored at −20°C.

### Problem 2

Low recovery of ssDNA from the polymer-rich phase (Step 2C).

### Potential solution

• Recovery of ssDNA is a time-sensitive step due to the dependency of droplets on fuel. Work quickly and strictly adhere to the indicated incubation times.
• The droplet pellet formed after centrifugation can be very small and transparent. To confirm the presence of a pellet, consider adding a dye-labelled RNA oligo (e.g., a Cy5-tagged RNA) at a concentration of ∼1 μM to the reaction mixture. This will enable identifying a pellet by fluorescence even in the absence of specific excitation of the dye molecule. The RNA concentration of the labelled RNA (for short oligonucleotides) is less than 1% of the total RNA concentration in the system and therefore should not significantly impact partitioning results. In addition, it will be digested by RNase in later steps, rendering this a non-issue for the subsequent application.
• If working with different droplet systems, ensure that your downstream clean-up protocol is compatible with the droplet material. Complex coacervates commonly contain poly-anions and poly-cations that can bind to the column material of spin-down columns from PCR clean-up kits. This may result in oversaturation of the column material due to its limited capacity and cause the loss of ssDNA material during loading and washing.

### Problem 3

Low ligation yields in the one-pot reaction with T4 DNA ligase and T4 PNK (Step 3I).

### Potential solution

• Depending on the system, carryover of droplet material may inhibit T4 DNA ligase. We found that poly-styrene sulfonate and dextran sulphate can both inhibit ligase activity, but they are difficult to separate from the ssDNA due to their poly-anionic character. It is paramount that the ssDNA is recovered cleanly from your droplet sample.
• If your library contains fixed ends (even just one or two nucleotides), the P5 and/or P7 splint oligos can be modified to match these regions. This will significantly increase yields, as more compatible splints will match your library design.

### Problem 4

The files for each individual sample are almost empty, with a large number of reads ending up in the ‘Undetermined’ file (Step 7E).

### Potential solution

• Confirm that the correct index sequence was entered in the correct orientation in the sample table of the sequencer.
• If demultiplexing on the sequencer fails, it is possible to demultiplex manually using cutadapt. Follow the instructions at: https://cutadapt.readthedocs.io/en/stable/guide.html#demultiplexing.
• Reducing the PhiX spike-in will reduce the number of ‘Undetermined’ reads, as all reads from the PhiX control will be stored here. However, going too low on the spike-in might result in poor cluster formation and low-quality sequencing data.

### Problem 5

The sequencing resulted in a low number of reads with a high occupancy but low pass filter on the sequencing flow cell (Step 7E).

### Potential solution

• This is most likely due to overloading of the flow cell. Verify the loaded concentration, ideally with an orthogonal method (e.g., using a TapeStation or a Qubit).
• Reduce the concentration loaded. We obtained good results with concentrations as low as 8 pM on the iSeq 100, despite the recommended loading concentration being between 50 pM and 200 pM.

## Resource availability

### Lead contact

Further information and requests for resources and reagents should be directed to and will be fulfilled by the lead contact, Hannes Mutschler (hannes.mutschler@tu-dortmund.de).

### Technical contact

Technical questions on executing this protocol should be directed to and will be answered by the technical contact, Corbin Machatzke (corbin.machatzke@tu-dortmund.de).

### Materials availability

This study did not generate new unique materials.

### Data and code availability

Original data generated using this method are available at https://doi.org/10.17877/TUDODATA-2025-MFE1EMF9. The published article includes all scripts and codes needed to generate the results presented in this work. They can be found in the main text of this work or at https://doi.org/10.5281/zenodo.17735401.

## Acknowledgments

The BoekhovenLab is grateful for support from the TUM Innovation Network – RISE, funded through the Excellence Strategy. This research was conducted within the Max Planck School Matter to Life, supported by the German Federal Ministry of Education and Research (BMBF) in collaboration with the Max Planck Society, and funded by the Deutsche Forschungsgemeinschaft (DFG, German Research Foundation) under Germany’s Excellence Strategy – EXC-2094 – 390783311. The BoekhovenLab is grateful for support from the European Research Council through ERC Starting Grant 852187 and ERC Consolidator Grant 101124380. H.M. is grateful for support from the European Research Council through the ERC Synergy Grant 101166888.

## Author contributions

C.M. worked on method design and development, as well as sample preparation, sequencing, and bioinformatic data analysis. A.-L.H. worked on sample preparation. J.B. and H.M. worked on conceptualizing the project. All authors worked together on writing and revising the manuscript. C.M. and A.-L.H. have contributed equally and have the right to list their name first in their CV or any bibliographical list.

## Declaration of interests

The authors declare no competing interests.

## References

[1] Machatzke C., Holtmannspötter A.-L., Mutschler H., Boekhoven J. (2025). DNA affects the phenotype of fuel-dependent coacervate droplets. Chemrxiv.

[2] Donau C., Späth F., Sosson M., Kriebisch B.A.K., Schnitter F., Tena-Solsona M., Kang H.-S., Salibi E., Sattler M., Mutschler H., Boekhoven J. (2020). Active coacervate droplets as a model for membraneless organelles and protocells. Nat. Commun..

[3] Späth F., Donau C., Bergmann A.M., Kränzlein M., Synatschke C.V., Rieger B., Boekhoven J. (2021). Molecular Design of Chemically Fueled Peptide-Polyelectrolyte Coacervate-Based Assemblies. J. Am. Chem. Soc..

[4] Bergmann A.M., Donau C., Späth F., Jahnke K., Göpfrich K., Boekhoven J. (2022). Evolution and Single-Droplet Analysis of Fuel-Driven Compartments by Droplet-Based Microfluidics. Angew. Chem. Int. Ed..

[5] Wenisch M., Li Y., Braun M.G., Eylert L., Späth F., Poprawa S.M., Rieger B., Synatschke C.V., Niederholtmeyer H., Boekhoven J. (2025). Toward synthetic life—Emergence, growth, creation of offspring, decay, and rescue of fuel-dependent synthetic cells. Chem.

[6] Holtmannspötter A.-L., Machatzke C., Begemann C., Salibi E., Donau C., Späth F., Boekhoven J., Mutschler H. (2024). Regulating Nucleic Acid Catalysis Using Active Droplets. Angew. Chem. Int. Ed..

[7] Mirlohi K., Blocher McTigue W.C. (2024). Coacervation for biomedical applications: innovations involving nucleic acids. Soft Matter.

[8] Frankel E.A., Bevilacqua P.C., Keating C.D. (2016). Polyamine/Nucleotide Coacervates Provide Strong Compartmentalization of Mg2+, Nucleotides, and RNA. Langmuir.

[9] Wollny D., Vernot B., Wang J., Hondele M., Safrastyan A., Aron F., Micheel J., He Z., Hyman A., Weis K. (2022). Characterization of RNA content in individual phase-separated coacervate microdroplets. Nat. Commun..

[10] Robertson M.P., Joyce G.F. (2012). The origins of the RNA world. Cold Spring Harb. Perspect. Biol..

[11] Orgel L.E. (1998). Polymerization on the rocks: theoretical introduction. Orig. Life Evol. Biosph..

[12] Horning D.P., Joyce G.F. (2016). Amplification of RNA by an RNA polymerase ribozyme. Proc. Natl. Acad. Sci. USA.

[13] Wochner A., Attwater J., Coulson A., Holliger P. (2011). Ribozyme-catalyzed transcription of an active ribozyme. Science.

[14] Troll C.J., Kapp J., Rao V., Harkins K.M., Cole C., Naughton C., Morgan J.M., Shapiro B., Green R.E. (2019). A ligation-based single-stranded library preparation method to analyze cell-free DNA and synthetic oligos. BMC Genom..

[15] (2015). https://www.bioinformatics.babraham.ac.uk/index.html.

[16] Ewels P., Magnusson M., Lundin S., Käller M. (2016). MultiQC: summarize analysis results for multiple tools and samples in a single report. Bioinformatics.

[17] Martin M. (2011). Cutadapt removes adapter sequences from high-throughput sequencing reads. EMBnet. J..

[18] Shen W., Sipos B., Zhao L. (2024). SeqKit2: A Swiss army knife for sequence and alignment processing. iMeta.

[19] Python Software Foundation (2025).

[20] McKinney W. (2010). Proceedings of the 9th Python in Science Conference (SciPy).

[21] Harris C.R., Millman K.J., van der Walt S.J., Gommers R., Virtanen P., Cournapeau D., Wieser E., Taylor J., Berg S., Smith N.J. (2020). Array programming with NumPy. Nature.

[22] Hunter J.D. (2007). Matplotlib: A 2D Graphics Environment. Comput. Sci. Eng..

[23] Lorenz R., Bernhart S.H., Höner Zu Siederdissen C., Tafer H., Flamm C., Stadler P.F., Hofacker I.L. (2011). ViennaRNA Package 2.0. Algorithm Mol. Biol..

[24] Hofacker I.L., Fontana W., Stadler P.F., Bonhoeffer L.S., Tacker M., Schuster P. (1994). Fast folding and comparison of RNA secondary structures. Monatsh. Chem..

[25] Hofacker I.L., Fekete M., Stadler P.F. (2002). Secondary structure prediction for aligned RNA sequences. J. Mol. Biol..

[26] Mathews D.H., Disney M.D., Childs J.L., Schroeder S.J., Zuker M., Turner D.H. (2004). Incorporating chemical modification constraints into a dynamic programming algorithm for prediction of RNA secondary structure. Proc. Natl. Acad. Sci. USA.

[27] Bailey T.L. (2021). STREME: accurate and versatile sequence motif discovery. Bioinformatics.

[28] Bailey T.L., Johnson J., Grant C.E., Noble W.S. (2015). The MEME Suite. Nucleic Acids Res..

[29] Tareen A., Kinney J.B. (2019). Logomaker: Beautiful sequence logos in python. Bioinformatics.

[30] Janssen S., Giegerich R. (2015). The RNA shapes studio. Bioinformatics.

